# From real-time adaptation to social learning in robot ecosystems

**DOI:** 10.3389/frobt.2023.1232708

**Published:** 2023-10-04

**Authors:** Alex Szorkovszky, Frank Veenstra, Kyrre Glette

**Affiliations:** ^1^ RITMO Centre for Interdisciplinary Studies in Rhythm, Time and Motion, University of Oslo, Oslo, Norway; ^2^ Department of Informatics, University of Oslo, Oslo, Norway

**Keywords:** social learning, evolutionary robotics, entrainment, central pattern generator, cultural evolution

## Abstract

While evolutionary robotics can create novel morphologies and controllers that are well-adapted to their environments, learning is still the most efficient way to adapt to changes that occur on shorter time scales. Learning proposals for evolving robots to date have focused on new individuals either learning a controller from scratch, or building on the experience of direct ancestors and/or robots with similar configurations. Here we propose and demonstrate a novel means for social learning of gait patterns, based on sensorimotor synchronization. Using movement patterns of other robots as input can drive nonlinear decentralized controllers such as CPGs into new limit cycles, hence encouraging diversity of movement patterns. Stable autonomous controllers can then be locked in, which we demonstrate using a quasi-Hebbian feedback scheme. We propose that in an ecosystem of robots evolving in a heterogeneous environment, such a scheme may allow for the emergence of generalist task-solvers from a population of specialists.

## 1 Introduction

It is by now a mainstream opinion in robotics and artificial intelligence that a properly intelligent machine can only come about by continually interacting with its environment through some forms of sensory perception-action loops (Pfeifer and Bongard, 2006; Zador et al., 2023). Such situated cognition is a prevalent goal in evolutionary robotics, where robots come to adapt to their environments and exhibit morphological intelligence (Doncieux et al., 2015).

Taking evolutionary robotics to its logical conclusion, the Autonomous Robot Evolution project presents a radically bottom-up approach to design and fabrication of diverse populations of robots with high degrees of autonomy (Hale et al., 2019). The approach is neatly broken down into three components: fabrication of a robot from a genotype, learning in the physical world, and finally “mature life” in which tasks are performed, performance is evaluated, and the robot’s morphology and/or controller is passed to the next-generation. This cycle has been termed the Triangle of Life (Eiben et al., 2013). Of these three stages, learning is currently the least well developed.

There are several reasons for a robot to learn during its lifetime, and not only benefit from its successful ancestors’ genetic material. The “reality gap” refers to controllers evolved *in silico* not behaving the same in the real world due to imperfections in the simulation (Jakobi et al., 1995). Imperfections in manufacture also imply a noisy genotype-to-phenotype mapping. This learning may not even necessarily be a small tweaking of parameters: a child robot resulting from mutations or crossover is likely to have a different number of sensor inputs and motor outputs to its parents, so the controller architecture often needs to be reconfigured or learned from scratch.

A controller archive is a natural solution to the problem of varying morphologies. This archives a high-fitness controller for each class of morphology (for example, a certain number of inputs and outputs) as a starting point for future child robots in this class (Le Goff et al., 2022). Controllers tuned during lifetime learning can then be passed down to compatible descendants. This approach resembles “quality diversity” schemes (Pugh et al., 2016) in that solutions occupying different parts of the space of solutions are preserved.

When considering a time-varying or heterogeneous environment, however, it is worth asking whether a certain learning and evolution scheme promotes specialists or generalists. Consider, for example, a task that can be solved by either stepping or hopping. If this is used to determine fitness, certain morphology-controller combinations will evolve to do either one or the other. Now, imagine a real-world task appears that requires some combination of stepping *and* hopping. The evolutionary solution in this case would most likely come from a child of one stepper and one hopper. As this is a new task, a new controller would need to be learned from scratch for the right combination of parents’ morphologies. Additionally, if the more complex task has been learned but is then absent for some time, catastrophic forgetting is likely to occur if it is not explicitly archived (McCloskey and Cohen, 1989).

One solution to generalist task-solving involves multi-objective evolutionary optimization (De Carlo et al., 2021), another common quality diversity technique. In this case the Pareto front will include both specialists closer to the edges favouring different fitness functions, and “jack-of-all-trades” solutions close to the middle. A learning stage can also be implemented to optimize specialized behaviours using multiple copies of the controller, which can be switched between (de Bruin et al., 2023). So, for example, a morphology that accommodates both stepping and hopping can learn a separate controller for each.

Here, we propose an alternative scheme, in which robots learn from each other instead of on their own. That is, we propose situated social learning of a variety of movement patterns from different “teacher” robots. We demonstrate a key component of the proposed learning method on a variety of controllers evolved using multi-objective optimization, as in de Bruin et al. (2023). In principle, the teacher and learner can exist in different regions of the Pareto front and have different morphologies. One advantage of this approach is that either specific behaviours can be human-defined as tasks, and selected upon, or behaviours can emerge spontaneously from the population if they are useful for survival. The latter is an example of open-ended evolution (Taylor, 2012), which by removing potential bounds on complexity intends to produce the “full generativity of nature” (Soros and Stanley, 2014).

A key to the success of the human species is precisely this kind of social learning, which can greatly enhance problem solving abilities (Herrmann et al., 2007) and the accumulation of knowledge and skills over time (Boyd et al., 2011). Not only does this accumulation take place “vertically,” from older to younger kin, but also “horizontally” across whole societies. Identifying the conditions in which genes and culture co-evolve, and the aspects of cognition that make it possible, are primary goals of dual inheritance theory, or biocultural evolution (Boyd and Richerson, 1985). This differs from the “memetic” approach influential in computer science (Neri and Cotta, 2012) in that it is based on behaviours rather than information, and is hence a more suitable framework for situated agents.

Culture, in this sense of knowledge, practice and artifacts preserved via non-genetic means across generations, is not only confined to those preserved through syntactical language. It also includes gesture, dance, vocal calls, music and tool-use transmitted through action imitation. Recent studies have shown evidence of social learning of several of these behaviours in non-human animals, indicating those that higher forms may be built upon (Whiten, 2021; Aplin, 2022). These basic forms of social learning involve copying of another agent’s behaviour, followed by its transformation into autonomous behaviour. The propagation of behaviour from agent to agent in this way is termed “cultural transmission” (Mesoudi and Whiten, 2008).

A behaviour, in our case, is communicated as a periodic rhythm via a series of impulses (for example, indicating swing-to-stance transitions). The “learner robot” first synchronizes to the input, a process known as rhythmic entrainment (Schachner et al., 2009), and then self-synchronizes to preserve the resulting motion pattern. Insofar as the frequency or pattern of ground contact indicates a behaviour, this form of communication allows specific behaviours to not be restricted to a particular area of morphological space.

We will first review current and potential generative approaches to social sensorimotor learning, and then demonstrate a scheme to achieve this in a diverse population of central pattern generator (CPG) based robots. The first ingredient of this process, CPGs with the ability to spontaneously entrain to periodic stimuli, has recently been achieved (Szorkovszky et al., 2023a). We will first demonstrate that Hebbian-like spike timing-dependent plasticity can be used to lock-in movement patterns achieved through synchronization. Using autocorrelation functions, we characterize both the diversification of movement patterns, as well as the cultural transmission of patterns from teacher to learner. Finally, we propose how this approach can be incorporated into a learning scheme for evolving robots.

## 2 Related work

### 2.1 Imitation and sensorimotor learning

A number of subfields of robotics and computational neuroscience have already successfully modelled aspects of social sensorimotor learning. For robot arms, learning from demonstration is a common technique, where operator training data are generalized into smooth dynamical systems with stable fixed points or limit cycles at desired positions in absolute or relative space (Ravichandar et al., 2020). Using extra dimensions in the dynamical system can even allow multiple overlapping limit cycles, such as clockwise and anticlockwise circles (Khoramshahi and Billard, 2019). Less common is robotic imitation and coordination of gestures from visual signals (Billard and Arbib, 2002; Arbib et al., 2014). However, much progress has been made in this area, and it has recently been proposed as a plausible means for open-ended evolution, particularly with conditions that encourage spontaneity (Ikegami et al., 2021).

Social and collective behaviours are also commonly studied using wheeled robots such as e-pucks. Often this is through directly copying controller parameters (Heinerman et al., 2015; Bredeche and Fontbonne, 2022). A notable exception is Winfield and Erbas (2011) in which robots attempted to copy each others’ trajectories based on their visual perception of their neighbours. This results in “noisy imitation” and hence increased variation in behaviours. Another interesting application of collective robotics is the use of synchronization to identify faulty neighbours (Christensen et al., 2009).

Another field of intense research is in vocal learning: the production and imitation of speech sounds with biophysical models. These studies use a variety of machine learning methods to maximize the match between perceived and produced sounds (Pagliarini et al., 2020), including reward-modulated spike timing dependent plasticity (Warlaumont and Finnegan, 2016). Early work in this area showed that a discrete set of vocal sounds can emerge in a self-organized fashion from mutual interaction (Oudeyer, 2005).

More general motor pattern learning is also an active topic in computational neuroscience. Here, large reservoirs of recurrent spiking neurons are commonly used to model the learning of arbitrary patterns in time, generally with feedback control of chaotic outputs (Sussillo and Abbott, 2009; Laje and Buonomano, 2013). This method takes advantage of such networks’ universalizability (Maass and Markram, 2004) and capacity for multifunctionality (Flynn et al., 2021).

### 2.2 CPG-based entrainment

For locomotion, robotic systems have been created that can adapt frequencies of movement to intrinsic body mechanics. In Buchli et al. (2006), CPG controller parameters were continuously modified according to phase-error feedback. In this case, once the feedback is turned off, the learned behaviour can be set in place by its new parameters. However, the need to employ a phase variable and to calculate an error signal limits the potential complexity of inputs.

Neuron-based CPGs, which due to their nonlinearity are faster and more flexible in their adaptation, have also been used in feedback loops to adapt to mechanical resonances in real time (Williamson, 1998; Iwasaki and Zheng, 2006). In the case of locomotion, force feedbacks can enable adaptation to physical environments, even with interconnections between CPG modules disabled (Thandiackal et al., 2021).

It has recently been demonstrated that self-organization of a locomotion CPG without feedback is sufficient for real-time entrainment to external rhythms, such as those transmitted through audio (Szorkovszky et al., 2023a). This can be seen as an embodied version of the “dynamic attending” approach to beat perception, which was proposed using abstracted nonlinear oscillators (Large and Jones, 1999). More broadly, this falls within the approach of exploiting self-organized nonlinear dynamics in order to generate complex behaviours (Steingrube et al., 2010; Husbands et al., 2021).

## 3 Materials and methods

### 3.1 Virtual robots

**FIGURE 1:**
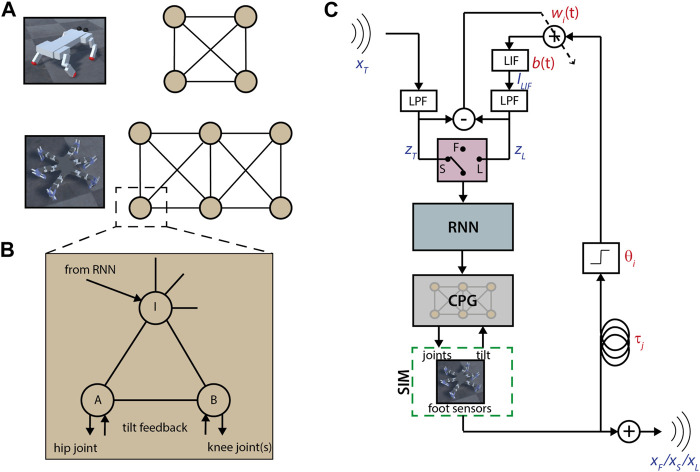
Robot controllers and learning scheme. **(A)** Quadruped and hexapod CPG layouts. Each circle is a module, connections are to the module’s interneuron. All connections between modules are inhibitory. **(B)** Diagram of a single CPG module. Each circle is a modified Matsuoka neuron. Connections with arrows are unidirectional, otherwise bidirectional. All connections can be inhibitory or excitatory. **(C)** Diagram of the learning scheme. Selection between free (F), synchronization (S) and learned feedback (L) stages is depicted as a switch. Variables shown in red are parameters, variables in blue are time series. The input signal *x*_*T*_ is the free output *x*_*F*_ of another agent. LPF: low pass filter; LIF: leaky integrate-and-fire neuron; RNN: recurrent neural network of 6 fully connected modified Matsuoka neurons with inhibition, thresholded so there is no output in the absence of input. During synchronization, *b*(*t*) = 1 and during the feedback stage, each *w*_*i*_(*t*) is fixed.

We begin with the same modular CPG and recurrent filter design as in Szorkovszky et al. (2023a). Each limb module contains three Matsuoka neurons modified to have input-dependent frequency. Two motor neurons drive the joints for each limb, while one interneuron is used for inter-limb coupling (see Figures 1A, B). The use of highly nonlinear CPGs and recurrent networks was intended to encourage self-organization and avoid fixed gait periods.

A constant input is applied to all neurons in a module, each with its own coefficient, to model the brain-stem modulation of the gait. In addition, external inputs are fed to the interneurons through a single-layer recurrent neural network (RNN) (see Figure 1C) consisting of six neurons of the same type as the CPG.

We used two virtual robot body layouts, simulated in Unity using the ML agents package (Grimminger et al., 2020). The first body type is a short-legged quadruped, based on the Open Dynamic quadruped (Juliani et al., 2018), and previously studied in Szorkovszky et al. (2023a). For this body, each motor neuron drives one joint angle (see Supplementary Material). Two control parameters were used to evaluate a controller’s flexibility: the first was the brain-stem drive, which typically affects gait period and amplitude. For the quadruped, an offset angle of the upper joint was also used to modulate the forward-backward direction of motion via the centre of mass.

The virtual hexapod body design is based on the 18-DOF robot used in Allard et al. (2022). Motor neuron output A was connected to the horizontal coxa joint, while output B was connected to both vertical joints, with two independent coefficients included in the genotype (see Supplementary Material). A simple coefficient for the coxa joint (−1 to 1) was used to modulate the direction of motion.

### 3.2 Evolution

The multi-objective evolutionary algorithm NSGA-III (Deb and Jain, 2013) was used to simultaneously optimize CPGs for flexibility in speed and direction, as well as stability, resulting in a diverse range of CPGs spanning the Pareto front. The genotype included CPG parameters, interconnection weights and tilt feedback weights [with the same ranges as in Szorkovszky et al. (2023a) for both morphologies] as well as central joint angles, angle limits and joint amplitude coefficients (see Supplementary Material of this paper for ranges).

Each evaluation was in three stages of 10 s each: 1) negative direction parameter with decreasing brain-stem drive, targeting fast backwards motion; 2) positive direction parameter with low and constant brain-stem drive, targeting steady forwards motion; and 3) positive direction parameter with increasing brain-stem drive, targeting fast forward motion. The fitnesses were calculated as.

$$F_{1}=-y_{1}-{\left(\frac{x_{1}}{x_{0}}\right)}^{2}$$ (1)

$$F_{2}=2y_{0}y_{2}-y_{2}^{2}-{\left(\frac{x_{1}}{x_{0}}\right)}^{2}$$ (2)

$$F_{3}=y_{3}-{\left(\frac{x_{1}}{x_{0}}\right)}^{2}$$ (3)

$$F_{4}=\frac{y_{0}^{2}H_{\text{tot}}}{1+t_{\text{tot}}}$$ (4)

where *y*_*i*_ and *x*_*i*_ were the parallel and perpendicular motion, respectively, in the *i*th stage. The perpendicular terms, with constant $x_{0}=\sqrt{5}$ m, were introduced to discourage turning. *F*_2_ is maximized at *y*_2_ = *y*_0_ = 2.5 m, while *F*_1_ and *F*_3_ are unbounded. The fourth fitness *F*_4_ targets stability across the entire evaluation, where *H*_tot_ is the mean height (normalized and limited for a maximum of 1), and *t*_tot_ is the root mean square body tilt.

Five runs were performed for each morphology, with each run using a population of 168 individuals evolving over 200 generations. After each run, four controllers were selected, each preferentially weighting one of the four fitness functions:

$$F_{m}^{*}=zF_{m}+\overset{4}{\underset{k=1}{\sum }}F_{k},$$ (5)

where *z* was incremented in intervals of one until the maximum of each $F_{m}^{*}$ was unique, or until a limit of *z* = 100 was reached.

For both morphologies, two of the post-evolution selections using Eq. 5 did not converge, meaning only three unique CPGs were output instead of four. Therefore, only 18 out of a possible 20 CPGs were selected for each, making a total of 36. For each of these CPGs, recurrent filter layers were evolved for maximum entrainment to a repetitive impulse pattern with a range of periods (Szorkovszky et al., 2023a).

### 3.3 Learning scheme

Using these 36 virtual robots, exhibiting a range of gait styles, we now consider firstly whether they can entrain to each others’ movement patterns, and secondly whether they can learned its entrained motion pattern. Entrainment ability has been demonstrated for various external periodic inputs in the quadruped morphology (Szorkovszky et al., 2023a; b). However, due to the open-loop nature of this entrainment, the robot’s original movement pattern reappears shortly after the input stops.

While reservoir-based methods can successfully learn to replicate time-series inputs using spike-timing dependent plasticity, as detailed in Section 2.1, these require large numbers of neurons to work effectively (Ganguli et al., 2008; Sussillo and Abbott, 2009). Instead, we approximate the outcome of a larger reinforcing feedback network by using time-delayed impulses from foot sensors to approximate the rhythmic input (see Figure 1C). These feedback connections are separate from the CPG and RNN modules, which are kept fixed and define the free motion and stimulus response, respectively. Therefore, the robot can return to its free motion by removing the feedback, or even switch between different learned motor patterns by interchanging the learned feedback parameters.

We consider every possible pairing of one “teacher,” which transmits its rhythmic step pattern, and one “learner” which attempts to entrain to the pattern and learn it. That is, each robot attempts to learn a new pattern from every other robot. The procedure for each teacher-learner pair consists of three stages, elaborated in the following sections. First, the learner synchronizes in an open-loop fashion to impulses from the teacher’s steps. Secondly, feedback parameters are learned to approximate the teacher’s input while still in a synchronized state. Finally, the teacher input is replaced with the feedback signal, and the learner then continues its behaviour autonomously, in a closed-loop “self-synchronized” state.

Foot sensors were added in simulation to record all instances of swing-to-stance transitions. Each of the 36 controllers was initially run for 60 s (4,000 frames at Δ*t* = 15 ms) to record its free motion, which is also its “teacher” output. This was the sum of impulses for each foot at each time step. The amplitude for each foot was one-half of the impulse amplitude used during evolution. In addition, the natural period of the robot’s free motion was calculated using autocorrelation of the CPG outputs.

Of the 36 controllers, five were discarded due to foot-dragging behaviour, resulting in an average of less than one step every two periods, and hence very low output levels. All teacher-learner combinations from the remaining 31 robots were run in the following three stages, each of 60 s duration.

### 3.4 Synchronization stage 1: feedback delay learning

The impulses in the teacher output were run through an exponential low-pass filter, using the decay rate in the learner’s genotype. This decay rate was optimized during evolution for entrainment to rhythmic impulse patterns. This input (*z*_*T*_) was then passed through the recurrent filter layer to the CPG.

After 60 s, a two-time cross-correlation was performed between each leg’s impulse output and the low-pass filtered input. All peaks were then identified with height greater than zero, with a distance of more than 1/20th of the learner’s CPG period from any higher peak, and with a lag of less than the learner’s CPG period.

The average number of foot sensor outputs per cycle was also calculated, and this was used as a threshold *θ*_*i*_ for limb *i*. This is because a synchronized system containing *n* teacher impulses and *m*_*i*_ foot steps per cycle for limb *i* is expected to generate *nm*_*i*_ cross-correlation peaks for each limb. Therefore, if *m*_*i*_ is greater than one, generating one feedback impulse per step will produce more than the *n* impulses in the input.

### 3.5 Synchronization stage 2: feedback weight learning

The second synchronization stage is simply a continuation of the first, but now the foot sensors with the calculated delays are fed into a leaky integrate-and-fire (LIF) neuron to combine them into a single feedback signal *z*_*L*_(*t*). Weights for the delayed impulses are learned continuously in order to match *z*_*L*_(*t*) to the input *z*_*T*_(*t*) as closely as possible.

The impulse signals per limb *S*_*i*_ are created using the delays *τ*_1_..*τ*_*p*_, where *p* is the number of cross-correlation peaks. and thresholds *θ*_*i*_ determined from the previous stage:

$$S_{i}\left(t\right)=I\left(\overset{p}{\underset{j=1}{\sum }}s_{i}\left(t-\tau_{j}\right)\geq \theta_{i}\right),$$ (6)

where *s*_*i*_(*t*) is 1 if the *i*th foot sensor is triggered during a three frame window centred at time *t*, and 0 otherwise, and I is an indicator function. A learner output *z*_*L*_(*t*) is then generated on-the-fly using the following update equation:

$$\Delta z_{L}\left(t\right)=I_{\text{LIF}}\left(t\right)-\gamma z_{L}\left(t\right),$$ (7)

where *γ* is the learner robot’s low pass filter decay parameter. We use a standard leaky integrate-and-fire output current *I*_LIF_ and membrane voltage *V*_LIF_, where

$$I_{\text{LIF}}\left(t\right)=\left\{\begin{array}{c} I_{\text{out}}\;\text{if}\;V_{\text{LIF}}\left(t-1\right)>b\left(t\right)\\ 0\;\text{otherwise}, \end{array}\right.$$ (8)

and

$$V_{\text{LIF}}\left(t\right)=\left\{\begin{array}{c} 0\;\text{if}\;V_{\text{LIF}}\left(t-1\right)>b\left(t\right)\\ \sum_{i}h\left(w_{i}\left(t\right)\right)S_{i}\left(t\right)+\left(1-\Gamma \right)V_{\text{LIF}}\left(t-1\right)\;\text{otherwise}. \end{array}\right.$$ (9)

Here, *h*(*x*) is a rectified linear unit, meaning that the inputs are strictly excitatory. The firing threshold *b*(*t*) is set to a constant value of one during the learning stage. For the results presented in this study, the LIF decay was set to Γ = 10 s^−1^ unless specified otherwise.

At the same time, the feedback weights are updated according to:

$$\Delta w_{i}\left(t\right)=\left\{\begin{array}{c} \frac{a}{m}\left(z_{T}\left(t\right)-z_{L}\left(t\right)\right)\;\sum_{t^{\prime }=0}^{T}e^{-\Gamma t^{\prime }\Delta t}S_{i}\left(t-t^{\prime }\right)\;\text{if}\;w_{i}\left(t-1\right)<w_{\text{max}}\\ 0\;\text{otherwise}, \end{array}\right.$$ (10)

where *a* = 0.01, *m* is the number of feet, *T* = 2/(Γ Δ*t*) rounded to the nearest integer, *w*_max_ is the maximum weight (set to 1.5 for this study), and the beginning weights *w*_*i*_(0) are zero for all *i*.

### 3.6 Feedback stage

In the final stage, the teacher input is replaced with the feedback from the feet governed by Eq. 7 with the feedback weights *w*_*i*_ fixed at their final learned values. Since stability cannot be guaranteed in the closed-loop case, we use a homeostatic feedback to stabilize the output level Husbands et al. (2021). We therefore adjust the LIF firing threshold *b*(*t*) if the feedback level is not close to the time-averaged teacher input E_*t*_[*z*_*T*_]:

$$\Delta b\left(t\right)=\left\{\begin{array}{c} \eta \left(\bar{z_{L}}-E_{t}\left[z_{T}\right]/k\right)\;\text{if}\;\bar{z_{L}}<E_{t}\left.\left[z_{T}\right]\right)/k\\ \eta \left(\bar{z_{L}}-kE_{t}\left[z_{T}\right]\right)\;\text{if}\;\bar{z_{L}}>kE_{t}\left.\left[z_{T}\right]\right)\\ 0\;\text{otherwise}, \end{array}\right.$$ (11)

where $\bar{z_{L}}$ is a moving average of the last 200 frames (3 s), *b*(0) = 1, *η* > 0 and *k* > 0. Hence, if too many foot sensors are triggered (for example, due to noise or imperfect feedback), producing excess input, the threshold is raised in order to lower the rate of firing of the LIF neuron. Likewise, if the robot is not stepping enough to produce the correct feedback level, the threshold is lowered. In this study, we use *k* = 1.5, so that there is a tolerance of 50% in the feedback level, and an increment *η* = 2 × 10^−4^.

### 3.7 Analysis

Analysis of the controllers’ similarity of output patterns was done using autocorrelation functions. The differences between movement patterns for the free, synchronized and learned (closed-loop feedback) trials were calculated using:

$$d\left(X,Y\right)=\frac{1}{T_{T}}\int_{0}^{T_{T}}{\left(R_{XX}\left(\tau \right)-R_{YY}\left(\tau \right)\right)}^{2}d\tau$$ (12)

where *X* and *Y* are two low-pass filtered time series, *T*_*T*_ is the CPG period of the teacher, and *R*_*xx*_(*τ*) is the autocorrelation of time-series *x* at lag *τ*, calculated over the last 40 s of each stage. This function, rather than the cross-correlation was used as it is relatively insensitive to relative phase drifts. Low-pass filters were used due to the inherent noisiness of correlation functions when one or both time series consist of short spikes.

The period of motion was also determined from the autocorrelation functions of the joint outputs. For each limb, a complex signal was made with a real part corresponding to the hip joint, and imaginary part corresponding to the knee joint, using the last 40 s of each stage. The real parts of the autocorrelation functions were then averaged, and the lag at the highest peak at *τ* > 0.1 s was used as the period, while its height was used as a measure of stability.

## 4 Results

We focused on two abilities. First is the ability of the learner to substantially modify its movement pattern temporarily through synchronization to a teacher, and/or in a lasting way through applied feedback. This ability implies a large synchronized-to-free difference *d*(*x*_*S*_, *x*_*F*_) and learned-to-free difference *d*(*x*_*L*_, *x*_*F*_), respectively. These were calculated for every teacher-learner pair.

Another important ability is to retain the input signal in the feedback, and then to transmit it with some fidelity. This can be quantified by the difference between the feedback signal and the input *d*(*z*_*L*_, *z*_*T*_) during the closed-loop feedback stage, and the difference between the teacher input and the final learner output that would be transmitted further *d*(*x*_*L*_, *z*_*T*_). Both of these are low for well performing pairs.

**FIGURE 2:**
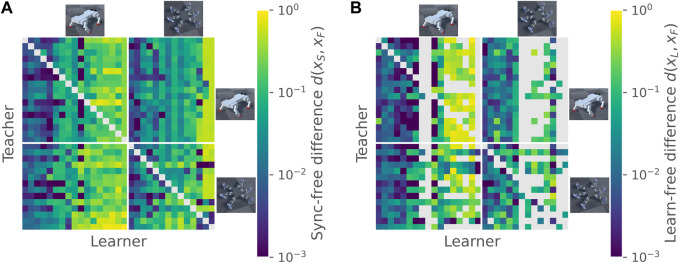
Diversification of gait pattern. Panel **(A)** shows the difference between synchronized and free gaits for all pairs of teachers and learners, ordered by morphology. Lower values indicate a smaller difference. Within each morphology, individuals are ordered by their mean synchronization difference as a learner. Diagonals are left blank since individuals were not tested against themselves in teacher-learner pairs. Panel **(B)** shows the difference between feedback-learned and free gaits, ordered as in **(A)**. Non-diagonal blank elements indicate that no cross-correlation peaks were found during the period learning stage, and hence the feedback stage was not run.

Synchronization of learner to teacher was largely learner-dependent, and did not show substantial preferences for the same morphology (see Figure 2A) or evolutionary run (see Supplementary Material for unsorted teacher-learner pair plots). Average within-teacher variability of the synchronized-to-free difference *d*(*x*_*S*_, *x*_*F*_) was 72% greater than average within-learner variability (standard deviation of 0.16 compared to 0.093). Although some learners rarely succeeded to proceed to the feedback stage (most often due to reduced motion), those that did synchronize successfully tended to also lock in new motion patterns in the feedback stage, as shown by Figure 2B. Here, the within-learner variability was more than twice the within-teacher variability (standard deviation of 0.176 compared to 0.087). In other words, some gaits are more able to be modified than others, while the characteristics of the teacher’s gait appear relatively unimportant.

**FIGURE 3:**
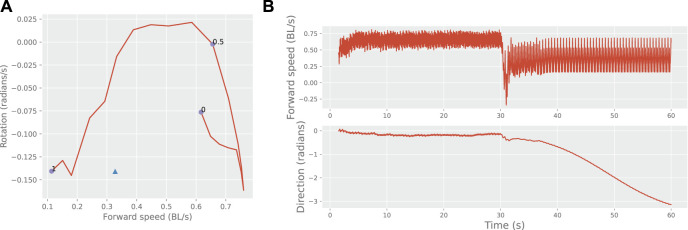
Behavioural switching. Panel **(A)** shows the mean movement characteristics of one learner during free motion as a function of the brain-stem input (line and circles), and the modified movement characteristics under self-synchronization (triangle). BL: body lengths. Panel **(B)** shows the learner’s switching from its intrinsic movement pattern (brain stem input 0.5) to its learned pattern upon the application of feedback parameters after 30 s.

Another way to show diversification is to examine movement characteristics such as speed and rotation. An example of a learner’s movement profile modified by the learned feedback is shown in Figure 3. In this example, the learned motion can be seen to be outside the normal range of motion patterns controlled by the brain-stem drive. Switching between the intrinsic and learned motion is possible by simply turning on the feedback.

**FIGURE 4:**
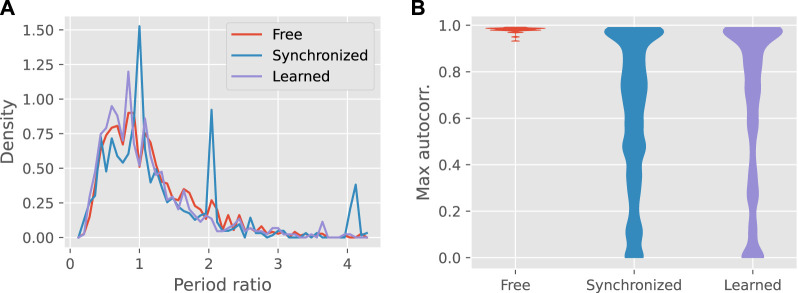
Panel **(A)** shows the frequency distribution of the ratio of the output to input period over all teacher-learner pairs, for the three input conditions. **(B)** Violin plots of the overall maximum of the autocorrelation function, showing the distributions over all teacher-learner pairs. The distribution for learned gaits only includes pairs for which feedback was actually applied.

The synchronized motion, as expected, often matched the period of the teacher, or a multiple thereof, as shown by Figure 4A. By comparison, robots with learned feedback tended to drift away from these periods, most often to a shorter one, and approached the input-free distribution.

Although feedback learning was less reliable than synchronization at copying the teacher’s gait period, learned controllers were more stable than the synchronized controllers, as shown by the height of the autocorrelation peak (see Figure 4B). Compared to synchronized gaits, learned gaits were more likely to have autocorrelation peaks near zero or one. The overall median for learned gaits was 0.76 compared to 0.66 for synchronized gaits (Mann-Whitney U-test: *p* < 0.001).

**FIGURE 5:**
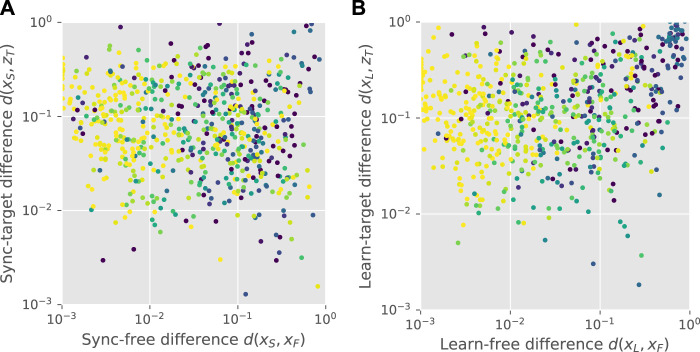
Cultural transmission. Panel **(A)** shows a scatter plot of the sync-free difference against the sync-target difference for all pairs, with colour indicating the autocorrelation peak height, ranging from zero (dark blue) to one (light yellow). Points on the lower-right correspond to synchronized gaits that are closer to the teacher’s gait than the learner’s original gait. Panel **(B)** shows the equivalent plot for learned gaits.

Upon both synchronization and feedback learning, agents that deviated more from their free gait were less stable, as shown by Figure 5. Some, however, had gait patterns closer to the input than their own free gait, shown by points on the lower-right of the plots (*d*(*x*_*S*_, *x*_*T*_) < *d*(*x*_*S*_, *x*_*F*_), and *d*(*x*_*L*_, *x*_*T*_) < *d*(*x*_*L*_, *x*_*F*_), respectively). A significant proportion of controllers in each stage were closer to the input pattern than the free pattern, as shown at the bottom right of each panel, which we call the “success rate.” Under synchronization, the learner was closer to the input for 43.5% of pairs. After feedback learning, the success rate was 23.7% among pairs where the feedback stage was run (total 15.7%). Hence, for some teacher-learner pairs, the combination of synchronization and feedback led to successful cultural transmission (see Supplementary Material for teacher-learner matrix plots). However, in other cases, the feedback led to a motion pattern unrelated to both the learner’s free motion and the teacher’s motion, as shown by the points in the top-right corner of Figure 5B. The low autocorrelation in this area implies that the feedback is leading to chaotic behaviour, and that the stabilization method can therefore be further optimized.

**FIGURE 6:**
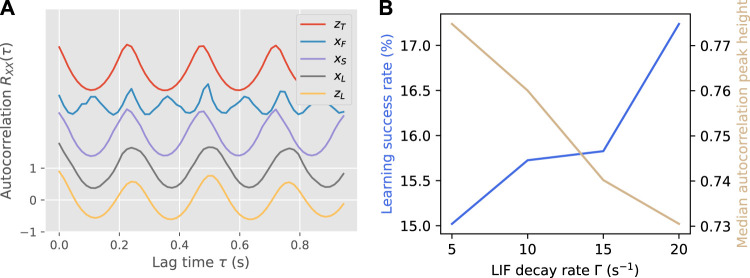
Panel **(A)** shows an example of autocorrelation functions modified by synchronization and learning, for the same teacher-learner pair as in Figure 3. Each autocorrelation function is offset from the previous by 1 for visibility. For this pair, *d*(*x*_*S*_, *x*_*F*_) = 0.279, *d*(*x*_*L*_, *x*_*F*_) = 0.240, *d*(*x*_*S*_, *z*_*T*_) = 0.002, *d*(*x*_*L*_, *Z*_*T*_) = 0.145. Panel **(B)** shows the learning success rate and median autocorrelation peak height over all teacher-learner pairs as a function of the LIF decay rate Γ.

An example of successful synchronization and learning is shown in Figure 6A. While the shape of the autocorrelation was largely kept upon feedback learning, there was a slight change in period that increased the learn-to-target difference.

The main parameter that was tuned was the LIF decay rate Γ, which determines the window in which impulses from different limbs can be combined. As shown in Figure 6B, there is a trade-off between stability and flexibility, with a shorter window (higher Γ) increasing the success rate of learning but decreasing the average stability of the final gaits. This illustrates the importance of accurate timing in the feedback, as an impulse from one foot that reaches the LIF neuron early or late will either reduce the accuracy of the input pattern (long window) or destabilize the gait completely (short window).

## 5 Discussion

We have demonstrated a proof of principle of a simple social learning scheme for robot gaits. Useful behaviours can be imitated by only communicating a series of foot contact events, such as via audible footsteps. Depending on fidelity, the imperfect copying that we demonstrate (Winfield and Erbas, 2011) can generate behavioural diversification and/or cultural transmission, which can be seen as population-level processes of exploration and exploitation, respectively.

Our scheme is a potential starting point towards robot to robot imitation in evolving populations where morphologies can differ drastically. Alternatives involving visual processing are computationally intensive and rely on an understanding of an agent’s own body and that it is imitating. New ways of communicating behaviour, either implicitly or explicitly, using rhythmic signals, would be a valuable continuation on this path.

The ability to entrain to a stimulus is a prerequisite of the demonstrated technique. However, the CPG architecture used here is modular, so can be straightforwardly implemented in the modular framework typically used in an evolutionary robotics setting Hale et al. (2019).

Our approach allows for multiple behaviours to be learned and switched between. Therefore, a learning environment could be composed of several elementary tasks, and individuals learn new tasks from teachers who have mastered them. We found that individuals differed substantially in their ability to learn new motion patterns, as quantified by the average difference between original patterns and those learned from various teachers. By incorporating the performance from numerous tasks into a fitness function, good generalist learners could therefore be selected by evolution.

Another possibility is for individuals to learn a behaviour-environment mapping, so that they automatically decide which motion pattern to use in an environment, and new individuals learn both the mapping and motion pattern from their neighbours. This unsupervised scheme avoids imposing artificial divisions in the learning environment and defining numerous tasks. Such an absence of designed goals could help to satisfy the theoretical requirements for the goal of open-ended evolution (Soros and Stanley, 2014). This lack of oversight, however, comes with risks of maladaptive behaviour spreading quickly, and may require precautionary safeguards (Eiben et al., 2021).

We found that on average, closed-loop feedback was more stable than the open-loop synchronization, despite pronounced delays in the feedback. Delayed feedback has been successfully used to generate a variety of motion patterns from discrete-time chaotic oscillators (Steingrube et al., 2010), and our results show this may also be a promising avenue for continuous-time CPGs with impulse feedback. The integrate-and-fire neuron with self-adapting threshold was a key factor in the stability of the learned gaits. The stability could further be increased using a more detailed spike-timing dependent plasticity (Kempter et al., 1999), which may be useful for adapting to different terrains.

Culture, like any human behaviour, is not trivial to generate in an artificial setting. However, there are clues pointing to the bootstraps that it is lifted by. It is believed that copying specifically via imitation of actions, as opposed to emulation of outcomes, is crucial for sustaining cultural transmission of complex behaviours (Whiten et al., 2009). To better understand how to sustain complexity, iterated learning over many individuals can be tested (Ravignani et al., 2016).

Our work can also inform research into the function of rhythmic entrainment. Due to the fact that animals that can entrain to a beat are often skilled at vocal mimicry, it has been widely theorized that these processes are built on the same neural substrate (Schachner et al., 2009). Forms of entrainment have also been linked to faculties for temporal prediction (Patel and Iversen, 2014), turn-taking (Takahashi et al., 2013), separating other agents from objects (Premack, 1990), and more advanced social capabilities such as joint attention (Knoblich and Sebanz, 2008).

In general, it is important to understand the link between rhythmic movement and cognition. Neuromorphic models of locomotion are potentially crucial for a bottom-up development of intelligence as they are dynamical systems that can exhibit attractor states, a proposed solution to the symbol grounding problem (Pfeifer and Bongard, 2006). Opening these systems to inputs from other networks and sensory data from the physical environment leads to “open dynamical systems,” which constantly adapt in their attractor landscapes (Hotton and Yoshimi, 2011; Beer and Williams, 2015). We believe that our proposal fruitfully extends this idea to social environments. It remains to be seen whether agents influencing each other through their basic motion can lead to the emergence of new forms of perception and action.

## Data Availability

The datasets presented in this study can be found in online repositories. The names of the repository/repositories and accession number(s) can be found below: https://github.com/aszorko/COROBOREES/tree/Paper4.
