# Supplementary material for: From real-time adaptation to social learning in robot ecosystems
Source: Front Robot AI. 2023 Oct 4;10:1232708. doi: 10.3389/frobt.2023.1232708 (PMC10584317; doi:10.3389/frobt.2023.1232708)
Supplement: Supplementary file 1 [file DataSheet1.PDF]

frontiers

From real-time adaptation to social learning in robot ecosystems: Supplementary Material

1 SUPPLEMENTARY INFORMATION

1.1 Simulation parameters

For both morphologies, the CPG used a time interval of $\Delta t = 7.5$ ms while the simulation's physics and communication with the CPG was done at intervals of 15 ms. At each communication time, the CPG received inputs processed from the body's tilt, and sent joint positions based on the changes in rectified motor neuron outputs since the previous simulation step:

$$\phi_i(t) = \phi_{0,i} + \phi_{\text{lim},i} \left[2S \left(\frac{2A_i d_i}{\phi_{\text{lim},i}} \frac{\Delta[u_A]^+}{\Delta t} + \frac{2B_i d_i}{\phi_{\text{lim},i}} \frac{\Delta[u_B]^+}{\Delta t} \right) - 1 \right] + \phi_{C,i} \tag{S1}$$

where $S(x)$ is a logistic function, u_A is the neuron A activation, u_B is the neuron B activation, $[\,]^+$ indicates a rectified linear unit and i indicates the joint.

Tables S1 and S2 provide the ranges for the above parameters. Single numbers indicate fixed parameters, ranges in normal font indicate evolving parameters (encoded by integers between 1 and 10), and ranges in bold font indicate direction control parameters that were swept during each evaluation during evolution, along with the brain stem drive (see Szorkovszky et al. (2022)). For the quadruped, $\phi_{C,\text{leg}}$ was used for direction control (by shifting the centre of mass forwards or backwards), while for the hexapod, d_{coxa} was used as for direction control (by determining the relative sign of coxa and leg amplitudes). During the post-evolution experiments detailed in the present article, these parameters were set to their maximum value.

1.2 Hexapod evolution

The hexapod design was based on an open-source 3D model by Allard et al. (2022). Each limb was divided into three rigid segments, each connected to a servo motor. Although the design includes shock absorber springs, this degree of freedom was not included in the simulation. The CPG evolution, CPG selection and filter evolution were performed identically as for the quadrupeds in Szorkovszky et al. (2022), only with a different direction control parameter as described above.

1.3 Unsorted teacher-learner pairs

Figure S1 shows the equivalent of Figure 2 in the main text, but without sorting by synchronization performance. Instead, evolutionary runs are grouped together. Although some runs were clearly better than others, a preference for related controllers from the same run (i.e. close to the diagonal) was not visible.

Figure S2 shows the same pairs as Figure S1, but instead plots the difference between the teacher's input and the learner's output. In this case, a lower difference indicates better performance. Again, a bias towards related controllers was not visible.

Table S1. Quadruped parameter ranges. All units apart from A_i,B_i and d_i are in degrees.

Parameter	Value / Range
$\phi_{0,\text{hip}}$	[2.7, 27]
$\phi_{\text{lim,hip}}$	90
$\phi_{\text{C,hip}}$	0
A_{hip}	0
B_{hip}	0
d_{hip}	0
$\phi_{0,\text{leg}}$	[4.5, 45]
$\phi_{\text{lim,leg}}$	90
$\phi_{\text{C,leg}}$	**[-1.35, 1.35]**
A_{leg}	[0.005, 0.05]
B_{leg}	0
d_{leg}	1
$\phi_{0,\text{knee}}$	-[7.2, 72]
$\phi_{\text{lim,knee}}$	90
$\phi_{\text{C,knee}}$	0
A_{knee}	0
B_{knee}	[0.005, 0.05]
d_{knee}	1

Table S2. Hexapod parameter ranges. All units apart from A_i,B_i and d_i are in degrees.

Parameter	Value / Range
$\phi_{0,\text{coxa}}$	0
$\phi_{\text{lim,coxa}}$	27
$\phi_{\text{C,coxa}}$	0
A_{coxa}	0
B_{coxa}	[0.01, 0.1]
d_{coxa}	**[-1, 1]**
$\phi_{0,\text{leg}}$	[-12.15, 12.15]
$\phi_{\text{lim,leg}}$	45
$\phi_{\text{C,leg}}$	0
A_{leg}	[-0.045, 0.045]
B_{leg}	0
d_{leg}	1
$\phi_{0,\text{knee}}$	[-12.15, 12.15]
$\phi_{\text{lim,knee}}$	45
$\phi_{\text{C,knee}}$	0
A_{knee}	[-0.045, 0.045]
B_{knee}	0
d_{knee}	1

REFERENCES

Allard, M., Smith, S. C., Chatzilygeroudis, K., Lim, B., and Cully, A. (2022). Online damage recovery for physical robots with hierarchical quality-diversity. *arXiv preprint arXiv:2210.09918*

Szorkovszky, A., Veenstra, F., and Glette, K. (2022). Central pattern generators evolved for real-time adaptation. *arXiv preprint arXiv:2210.08102*

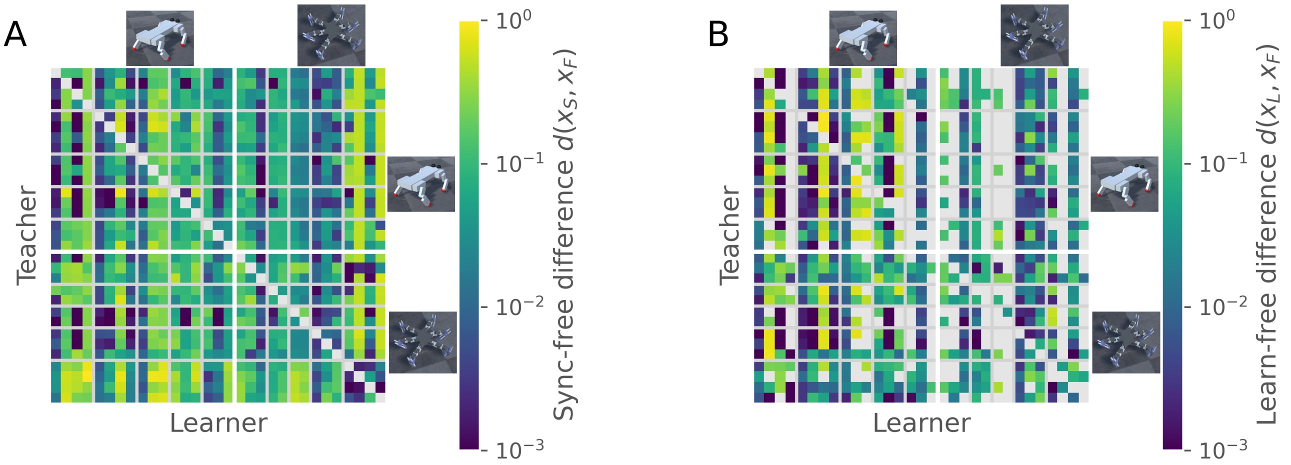

Figure S1. Differences from free gait pattern for unsorted teacher-learner pairs. Panel A shows the difference between synchronized and free gait patterns for all pairs of teachers and learners, ordered by morphology (divided by thicker white lines) and CPG evolution run (divided by thin grey lines). Panel B shows the difference between feedback-learned and free gaits, ordered as in (A). Non-diagonal blank elements indicate that no cross-correlation peaks were found during the period learning stage, and hence the feedback stage was not run.

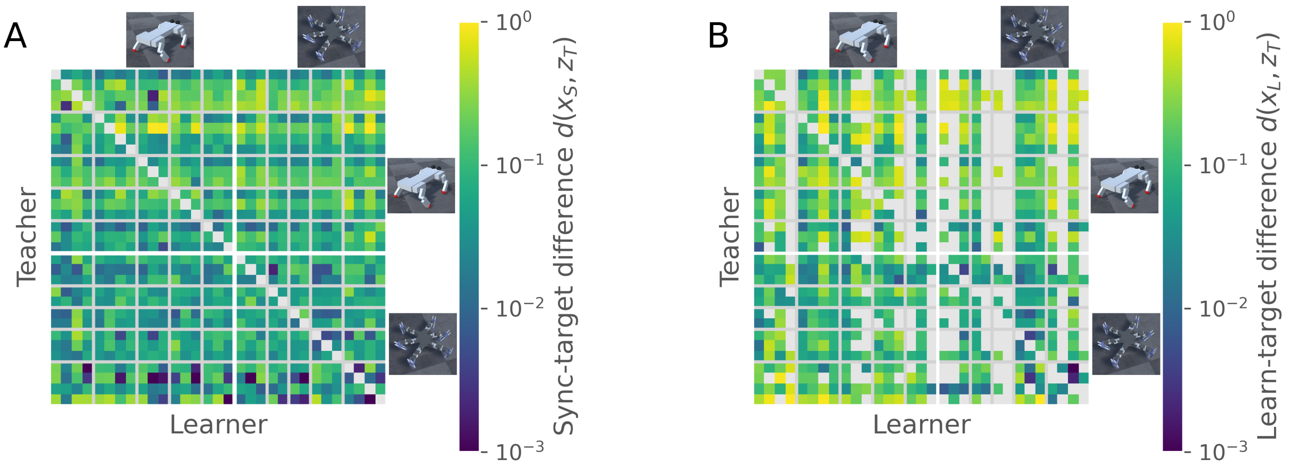

Figure S2. Differences from teacher input pattern for unsorted teacher-learner pairs. Panel A shows the difference between synchronized gait pattern and teacher input pattern for all pairs of teachers and learners, ordered by morphology (divided by thicker white lines) and CPG evolution run (divided by thin grey lines). Panel B shows the difference between feedback-learned and teacher input patterns, ordered as in (A). Non-diagonal blank elements indicate that no cross-correlation peaks were found during the period learning stage, and hence the feedback stage was not run.
